# High-throughput phenotyping of physiological traits for wheat resilience to high temperature and drought stress

**DOI:** 10.1093/jxb/erac160

**Published:** 2022-04-21

**Authors:** Pedro M P Correia, Jesper Cairo Westergaard, Anabela Bernardes da Silva, Thomas Roitsch, Elizabete Carmo-Silva, Jorge Marques da Silva

**Affiliations:** BioISI – Biosystems & Integrative Sciences Institute, Faculdade de Ciências da Universidade de Lisboa, Campo Grande, 1749-016 Lisboa, Portugal; Department of Plant and Environmental Sciences, Section of Crop Science, Copenhagen University, Højbakkegård Allé 13, 2630 Tåstrup, Denmark; BioISI – Biosystems & Integrative Sciences Institute, Faculdade de Ciências da Universidade de Lisboa, Campo Grande, 1749-016 Lisboa, Portugal; Department of Plant and Environmental Sciences, Section of Crop Science, Copenhagen University, Højbakkegård Allé 13, 2630 Tåstrup, Denmark; Department of Adaptive Biotechnologies, Global Change Research Institute, CAS, 603 00 Brno, Czech Republic; Lancaster Environment Centre, Lancaster University, Library Avenue, Lancaster LA1 4YQ, UK; BioISI – Biosystems & Integrative Sciences Institute, Faculdade de Ciências da Universidade de Lisboa, Campo Grande, 1749-016 Lisboa, Portugal; CSIRO Agriculture and Food, Australia

**Keywords:** Carbohydrate metabolism, climate change, drought resilience, food security, high temperature, high-throughput plant phenotyping, multispectral imaging, *Triticum aestivum*, water deficit, wheat

## Abstract

Interannual and local fluctuations in wheat crop yield are mostly explained by abiotic constraints. Heatwaves and drought, which are among the top stressors, commonly co-occur, and their frequency is increasing with global climate change. High-throughput methods were optimized to phenotype wheat plants under controlled water deficit and high temperature, with the aim to identify phenotypic traits conferring adaptative stress responses. Wheat plants of 10 genotypes were grown in a fully automated plant facility under 25/18 °C day/night for 30 d, and then the temperature was increased for 7 d (38/31 °C day/night) while maintaining half of the plants well irrigated and half at 30% field capacity. Thermal and multispectral images and pot weights were registered twice daily. At the end of the experiment, key metabolites and enzyme activities from carbohydrate and antioxidant metabolism were quantified. Regression machine learning models were successfully established to predict plant biomass using image-extracted parameters. Evapotranspiration traits expressed significant genotype–environment interactions (G×E) when acclimatization to stress was continuously monitored. Consequently, transpiration efficiency was essential to maintain the balance between water-saving strategies and biomass production in wheat under water deficit and high temperature. Stress tolerance included changes in carbohydrate metabolism, particularly in the sucrolytic and glycolytic pathways, and in antioxidant metabolism. The observed genetic differences in sensitivity to high temperature and water deficit can be exploited in breeding programmes to improve wheat resilience to climate change.

## Teaser

High-throughput phenotyping highlighted the importance of canopy architecture to fine-tune transpiration. The interplay between sucrolytic and glycolytic pathways is essential to tolerate drought at high temperature in wheat.

## Introduction

Wheat is a major staple food in numerous regions worldwide (FAOSTAT, 2019). Around 40% of the global wheat yield fluctuations are explained by environmental constraints, with heatwaves and drought being among the top stressors (Deryng *et al.*, 2014; Zampieri *et al.*, 2017). Moreover, several wheat-growing regions are characterized by hot and dry summers, where drought is likely to coincide with elevated temperatures more frequently in the future (Araus, 2002; Guerreiro *et al.*, 2018). Understanding the interaction effect between drought and high temperature on crop production is challenging, as simultaneous stresses may have additive, synergistic, or antagonistic effects on a plant’s physiology (Rizhsky *et al.*, 2004; Tricker *et al.*, 2018). Furthermore, such a response integrates several phenological and physiological processes under multigenic controls, and depends on the individual’s sensitivity to the microenvironment (Parent *et al.*, 2017). As a result, continuous monitoring of a plant’s response to controlled stress conditions is essential to understand plant–environment interactions. High-throughput phenotyping methods appeared to be the solution to compensate the otherwise labour-intensive and time-consuming classic methods of systematic plant phenotyping (Fiorani and Schurr, 2013; Fahlgren *et al.*, 2015; Tardieu *et al.*, 2017; Roitsch *et al.*, 2019).

Crop production is intimately dependent on carbon uptake by photosynthesis, and stomatal opening is vital for carbon capture. However, in response to water shortage, higher plants close stomata to limit water losses by transpiration (Chaves *et al.*, 2003; Nunes *et al.*, 2009; Duque *et al.*, 2013) and, when subjected to high temperatures, plants usually use evaporative cooling to reduce leaf temperature, which could otherwise be detrimental to several physiological processes (Carmo-Silva *et al.*, 2012; Costa *et al.*, 2013; Lawson *et al.*, 2018). An optimal balance between risk avoidance and performance is critical to crop production under water deficit and high-temperature conditions (Tardieu, 2012; Moshelion, 2020). When photosynthetic performance and plant growth are challenged by water shortage and elevated temperatures, remobilization of carbohydrate metabolism through adjustment of source–sink relationships is crucial to tolerate stress and accelerate recovery to reduce yield fluctuations (El Habti *et al.*, 2020; Correia *et al.*, 2021).

Several carbohydrate metabolism enzymes demonstrated fundamental functions in drought stress tolerance (Albacete et al., 2011, 2015; Pinheiro and Chaves, 2011; Secchi and Zwieniecki, 2016; Cuesta-Seijo *et al.*, 2019; Shokat *et al.*, 2020), although little is known about their role in drought at high temperature. Furthermore, stress exposure usually leads to excessive accumulation of reactive oxygen species (ROS), causing damage to plasma membranes, proteins, and pigments, and, if the capacity of scavenging and repairing mechanisms is exceeded, photosynthesis and crop performance are constrained (Foyer and Noctor, 2005; Tricker *et al.*, 2018). ­Antioxidant capacity was associated with tolerance to drought or heat stress in wheat (Sairam *et al.*, 2000; Lascano *et al.*, 2001; Zhang *et al.*, 2017), although the importance of ROS scavenging, enzymatically and/or by the production of several antioxidant compounds, in combined stresses is unknown.

In this study, we continuously monitored biomass allocation, plant–water relations, and reflective properties in 10 genotypes with heterogeneous performance under water deficit and high-temperature conditions. We included some of the best available elite advanced lines (check lines), selected based on their superior performance and verified in multiyear yield trials at the International Maize and Wheat Improvement Center (CIMMYT) breeding site (Reynolds *et al.*, 2017). Physiological trait lines (PT lines), the outline of a breeding strategy where crosses were designed to complement ‘source’ (biomass) with ‘sink’ traits (harvest index, kernel weight, grains per spike) (Reynolds *et al.*, 2017), and parental lines used in trait-based crossing of PT lines (Reynolds *et al.*, 2007; Manès *et al.*, 2012). The contrasting levels of tolerance to drought and heat of the 10 wheat genotypes were explored to (i) optimize high-throughput methods to phenotype wheat plants under drought and high temperature; (ii) profile the plant–water–environment relationship of each genotype under these conditions; and (iii) understand the regulatory mechanisms of the primary carbohydrate and antioxidant metabolism associated with a plant’s response to drought at high temperature. The effects of long-term (7 d) growth under high temperature (WW38) and water deficit at high temperature (WD38) on traits related to leaf reflectance proprieties, water use, and biomass accumulation were assessed in a high-throughput phenotyping station and linked to the regulation of carbohydrate and antioxidant metabolism.

## Materials and methods

### Germplasm and growth conditions

Ten spring wheat (*Triticum aestivum* L.) genotypes were selected based on their heterogeneous performance under water deficit or high-temperature conditions (Table 1). BORLAUG, SOKOLL, and BAJ are check lines; PASTOR and SOKWB_1 were included in the Stress Adapted Trait Yield Nurseries trial (SATYN, CIMMYT) and performed well in drought-stressed areas (SATYN series with odd numbers); PUBWB and SOKWB_2 showed outstanding performance in SATYN under heat stress conditions (SATYN series with even numbers); CMH82 and KSPA are parental lines; and PARAGON is a traditional UK spring wheat elite cultivar (Moore, 2015; Pennacchi *et al.*, 2019), with good tolerance to water deficit and high temperature when characterized alongside SOKOLL (Correia *et al.*, 2021).

Plants were grown from seeds for 15 d (DAS 15) in 1 litre well-watered pots containing horticultural substrate (SW Horto AB, Hammenhög) plus 10% perlite in a greenhouse at 25/18 °C (day/night), 50% relative humidity, and a photoperiod of 16 h. Plants were then moved to the phenotyping greenhouse (PhenoLab, PLEN UCPH), automated for plant care and movement, with the same climatic conditions, and were well watered (WW >90% field capacity). After 15 d (30 DAS), the temperature was changed to 38/31 °C (day/night), and the plants were randomly assigned to two different irrigation treatments: five plants per cultivar were maintained well watered (WW38), and five plants were subject to water deficit (WD38) for 7 d. WD was established by withholding watering and sustaining a minimum of 30% field capacity. The soil water content was determined gravimetrically by automatically weighing the pots twice a day, and irrigation was provided to compensate for evapotranspiration and keep the soil water content in the WW and WD pots. Pots containing only soil were maintained under the same watering regime and weighed to estimate non-transpirational water loss under WW38 and WD38 treatments. Leaf samples for biochemical analyses were collected at the end of the experiment (DAS 37), 5–7 h after the beginning of the photoperiod, frozen in liquid nitrogen, and stored at –80 °C.

### High-throughput data acquisition and extraction

Multispectral images were automatically acquired by a top-mounted CCD camera (PhenoLab, Videometer) twice a day from 15 to 37 DAS. A hemisphere set-up (PhenoLab, Videometer) was used to ensure homogeneous and diffuse illumination of the plants by high-power narrow-banded LEDs at 10 discrete spectral bands (365, 460, 525, 570, 645, 670, 700, 780, 890, and 970 nm). Thermography was obtained using a thermal camera (Flir A65, FLIR Systems Inc.). Multispectral images consisting of stacked photos, each with a specific spectral band, were analysed via a supervised classification method (normalized Canonical Discriminant Analysis, nCDA) in the software VideometerLab (version 3.0.33, Videometer). Based on this procedure, crop coverage (plant exposed area) was automatically calculated from segmented transformed images and pixel reflectance intensities extracted from the same region of interest (ROI). Thermographic data extraction was automated by using masks from the segmentation of multispectral images. In the software MATLAB (version r2019a, The MathWorks Inc.) the multispectral image was geometrically transformed by image registration to the thermal image. The resulting transformation file was used for automated transformation of all the multispectral masks to fit the geometry of the thermal images, allowing for segmentation of the thermal signal from each acquisition of the plant parts and calculation of the temperature statistics. Evapotranspiration was calculated from the gravimetric data obtained at the time of irrigation as the water loss per hour between consecutive measurements (mg H_2_O h^–1^). As all the pots in each treatment were in the same conditions (soil, capacity, and water availability), randomly distributed, automatically reorganized (four times daily), and measured twice a day, differences in evapotranspiration between them can be attributed to variations in plant transpiration.

### Plant harvesting and biomass prediction

At the end of the experiment (37 DAS), plants were harvested to measure aboveground and root FW and DW. Roots were separated from the soil by manual washing with water, and shoots were harvested by cutting immediately above ground level. FW was directly measured on an electronic scale, and DW was measured after oven-drying samples at 70 °C for 52 h. Architectural traits such as the number of leaves and tillers were also assessed before harvesting. Predictive models were constructed based on 16 machine learning regression methods implemented in the predMod module of the HTPmod Shiny application (Chen *et al.*, 2018a) and tested to predict plant biomass (FW and DW) from image-extracted features (37 DAS), as applied in Chen *et al.* (2018b). A 10-fold cross-validation strategy was adopted to check the prediction power of each regression model. The dataset was randomly divided into a training set (70% of plants) and a testing set (30% of plants). The trained model, based on the training data, was then applied to predict biomass for the testing set of plants. Models were considered for further application if the following criteria for the prediction performance were satisfied: (i) Pearson ­correlation coefficient of determination (*R*^2^) between the predicted values and the observed values >0.7; (ii) root mean squared relative error of cross-validation (MRSRE) <0.3; and (iii) predictive bias between the predicted and observed values (μ) <0.05. A Linear Support Vector Machine model (svm-linear, caret R package; Kuhn, 2008) was then used to predict FW and DW from 15 to 37 DAS from image-derived features.

### Growth modelling

Ten different mechanistic models implemented in the growMod module of the HTPmod Shiny application (Chen *et al.*, 2018a) were tested to model plant growth and applied as described in Chen *et al.* (2014). Models were considered if the following criteria for the fitting quality were satisfied: (i) *R*^2^>0.7 and (ii) *P*<0.05. The time point of maximum biomass (Timemax) and maximum final vegetative biomass (biomass at Timemax) were extracted from the models.

Intrinsic growth rate (GR), which measures the speed of growth, was estimated as:


$$\begin{array}{l} GR=\ln\left(Biomass\;at\;Timemax-Biomass\;at\;T1\right)/\left(Timemax-T1\right), \end{array}$$ (1)

where T1 is the time point of stress imposition (30 DAS).

The water use efficiency (WUE) from 30 to 37 DAS of each plant, corresponding to the biomass produced per water transpirated, was calculated as:


$$\begin{array}{l} WUE=\left(Biomass\;at\;Timemax-Biomass\;at\;T1\right)/\left(Timemax\;H_{2}O-T1H_{2}O\right), \end{array}$$ (2)

where T1H_2_O is the total water supplied at the time point of stress imposition, Timemax H_2_O is the total water supplied at Timemax, excluding the non-transpirational water loss (water loss in pots only with soil under the same irrigation regime). Comparison of plant growth between WW38 and WD38 conditions was assessed through a number of specific stress tolerance indices (Fischer and Maurer, 1978; Bouslama and Schapaugh, 1984; Grzesiak *et al.*, 2012) calculated with the parameters extracted from the growth models:


$$\begin{array}{l} Mean\;productivity=\left(Biomass\;at\;Timemax_{WW38}+Biomass\;at\;Timemax_{WD38}\right)/2 \end{array}$$ (3)

$$\begin{array}{l} Biomass\;reduction=Biomass\;at\;Timemax_{WD38}/Biomass\;at\;Timemax_{WW38} \end{array}$$ (4)

$$\begin{array}{l} Inflection\;point\;stability=Timemax_{WW38}Timemax_{WD38} \end{array}$$ (5)

$$\begin{array}{l} GR\;ratio=GR_{WD38}/GR_{WW38} \end{array}$$ (6)

$$\begin{array}{l} WUE\;ratio=WUE_{WD38}/WUE_{WW38} \end{array}$$ (7)

### Enzyme extraction

Frozen wheat leaf samples were homogenized in liquid nitrogen with 1% (w/v) insoluble polyvinylpyrrolidone (PVP). Total protein was extracted from samples (0.5 g FW) in ice-cold extraction medium containing 40 mM Tris–HCl pH 7.6, 3 mM MgCl_2_, 1 mM EDTA, 0.1 mM phenylmethylsulfonyl fluoride (PMSF), 1 mM benzamidine, 14 mM β-mercaptoethanol, and 24 μM NADP^+^, following the protocol described in Jammer *et al.* (2015). Total soluble protein (TSP) content was determined according to the Bradford method (Bradford, 1976) using BSA Fraction V as standard protein. Extracts were aliquoted, frozen in liquid nitrogen, and stored at –20 °C.

### Activity of carbohydrate metabolism enzymes

The activity of nine enzymes from carbohydrate metabolism was measured in thawed leaf extracts using a semi-high-throughput protocol in 96-well microtitre plates as described in Jammer *et al.* (2015) with variable extract volume (1–5 µl), and monitoring absorbance at 30 °C (ELx808, BioTek Instruments, Inc.). Briefly, aldolase (Ald; EC 4.1.2.13) and phosphofructokinase (PFK; EC 2.7.1.11) activities were measured by coupling the reactions with a glycerol-3-phosphate dehydrogenase (GPDH) NADH-dependent reaction and recording the decrease in absorbance at 340 nm due to conversion of NADH to NAD^+^. ADP-glucose pyrophosphorylase (AGPase; EC 2.7.7.27), glucose-6-phosphate dehydrogenase (G6PDH; EC 1.1.1.49), hexokinase (HXK; EC 2.7.1.1), phosphoglucoisomerase (PGI; EC 5.3.1.9), and phosphoglucomutase (PGM; EC 5.4.2.2) activities were assayed by linking the reactions with a G6PDH NADP-dependent reaction and recording the increase in absorbance at 340 nm due to conversion of NADP^+^ to NADPH. Cytoplasmic invertase (cytINV; EC 3.2.1.26) and vacuolar invertase (vacINV; EC 3.2.1.26) activities were measured in an end-point assay, and the amount of liberated glucose was determined by measurements of absorbance at 405 nm. All reactions were carried out in triplicate alongside control reactions (in the absence of substrate), and enzyme activity was expressed relative to the amount of TSP in each sample.

### Quantification of carbohydrates

Carbohydrates were extracted from frozen leaf samples homogenized in liquid nitrogen (0.1 g FW) by homogenization in ethanol (80% v/v) for 5 min at 80 °C and then centrifuged at 20 000 *g* for 5 min. The supernatant was collected, allowed to evaporate overnight at 60 °C, diluted in dH_2_O, and used to quantify sucrose, glucose, and fructose by an enzymatic method (kit AK00201, NZYTech) in a miniaturized protocol in 96-well microtitre plates following the manufacturer’s recommendations. The pellet was used to quantify starch by the same method after acidic hydrolysis in 30% HCl (v/v) at 90 °C for 15min.

### Activity of antioxidant enzymes

The activity of four enzymes from antioxidant metabolism was measured in thawed samples using a semi-high-throughput protocol in 96-well microtitre plates as described in Fimognari *et al.* (2020), with variable extract volume (1–5 µl), by monitoring absorbance at 30 °C (ELx808, BioTek Instruments, Inc.). Briefly, ascorbate peroxidase (APX; EC 1.11.1.11) activity was measured by recording oxidation of ascorbate at 290 nm. Catalase (CAT; EC 1.11.1.6) activity was assayed by recording the decomposition of H_2_O_2_ at 240 nm. Peroxidase (POX; EC 1.11.1.7) activity was measured by recording the formation of tetraguaiacol at 450 nm. Superoxide dismutase (SOD; EC 1.15.1.1) activity was measured by recording the inhibition of the oxidation of cytochrome *c* at 550 nm. All reactions were carried out in triplicate alongside control reactions (in the absence of substrate), and enzyme activity was expressed relative to the amount of TSP.

### Quantification of antioxidant capacity and metabolites

Antioxidant metabolites were extracted from frozen leaf samples homogenized in liquid nitrogen (0.4 g FW) by homogenization in pure methanol overnight in the dark at 4 °C and then centrifuged at 20 000 *g* for 5 min. The supernatant was aliquoted and stored at –20 °C. Antioxidant metabolites and capacities were quantified in thawed samples using a miniaturized protocol in 96-well microtitre plates and monitoring absorbance (Synergy HTX, BioTek Instruments Inc.). Trolox equivalents antioxidant capacity (TEAC) and ferric reducing antioxidant power (FRAP) were quantified as described in Correia *et al.* (2021). Total phenolic content was determined by the Folin–Ciocalteu method (Singleton *et al.*, 1999) by recording the absorbance at 765 nm. Galic acid standards in ddH_2_O were measured alongside the samples and used to prepare the respective calibration curves. The total phenolic content of the extract was expressed as mg galic acid equivalents per g of leaf (mg g^–1^ FW). The total flavonoid content was determined by the AlCl_3_ method (Zhishen *et al.*, 1999) by recording the absorbance at 510 nm. Catechin was used as a standard for the calibration curve. The total flavonoid content of the extract was expressed as mg catechin equivalents per g of sample (mg g^–1^ FW). Anthocyanin content was assessed by the pH-differential method (Giusti and Wrolstad, 2001) and expressed as cyanidin-3-glucoside equivalents (mg g^–1^ FW).

### Data pre-processing and analysis

Traits extracted from the images and gravimetric measurements were pre-processed for outlier detection, trait reproducibility, and collinearity assessment using the same approach as Chen *et al.* (2014). Briefly, Grubbs’ test (Grubbs, 1950) was used to detect outliers in replicated plants in each genotype under the same conditions and at the same data point, outliers were deleted, and missing values were imputed (missForest R package, Stekhoven and Bühlmann, 2012). Traits were considered as highly reproducible if the following criteria were satisfied in at least one treatment condition: (i) the median correlation coefficient over genotypes was >0.7; and (ii) the coefficients were significantly higher in replicates than in random plant pairs (Welch’s *t*-test *P*<0.05). To reduce the excessive correlation among explanatory variables (multicollinearity), a stepwise variable selection using variance inflation factors (VIFs) was applied; traits were considered if the VIF was <5. The observed variance in phenotypic traits, enzymatic activities, and amounts of metabolites was partitioned into components attributable to different sources of variation, the variation of genotype (G), environment (E), and their interaction (G×E), using the same approach as Chen *et al.* (2014). Briefly, a linear model was applied to determine the likelihood of genotype to phenotype linkage for each trait measured on each day, *P-*values were corrected for multiple comparisons with the Benjamini–Hochberg false discovery rate method (FDR), and the LOD scores (logarithm of odds) were calculated, as the -log probability (corrected *P*-value, FDR) (Joosen *et al.*, 2013). A heat map representation and hierarchical clustering were applied to the matrix of LOD scores or tolerance indices. ANOVA and post-hoc comparison (Duncan test, *P*<0.05) were also used to dissect the statistical significance of individual trait variation between genotypes. A *t*-test (*P*<0.05) was used for comparison between two treatments (WD38 and WW38) within the same genotype. A partial least squares discriminant analysis (PLS-DA) was performed to classify and discriminate plants into treatments (WW38 and WD38) and genotypes for each treatment, and to identify the key variables that drive such discrimination (MixOmics R package, Rohart *et al.*, 2017).

## Results

### Phenotypic descriptors reflecting the response to water deficit and high temperature

**Fig. 1.**
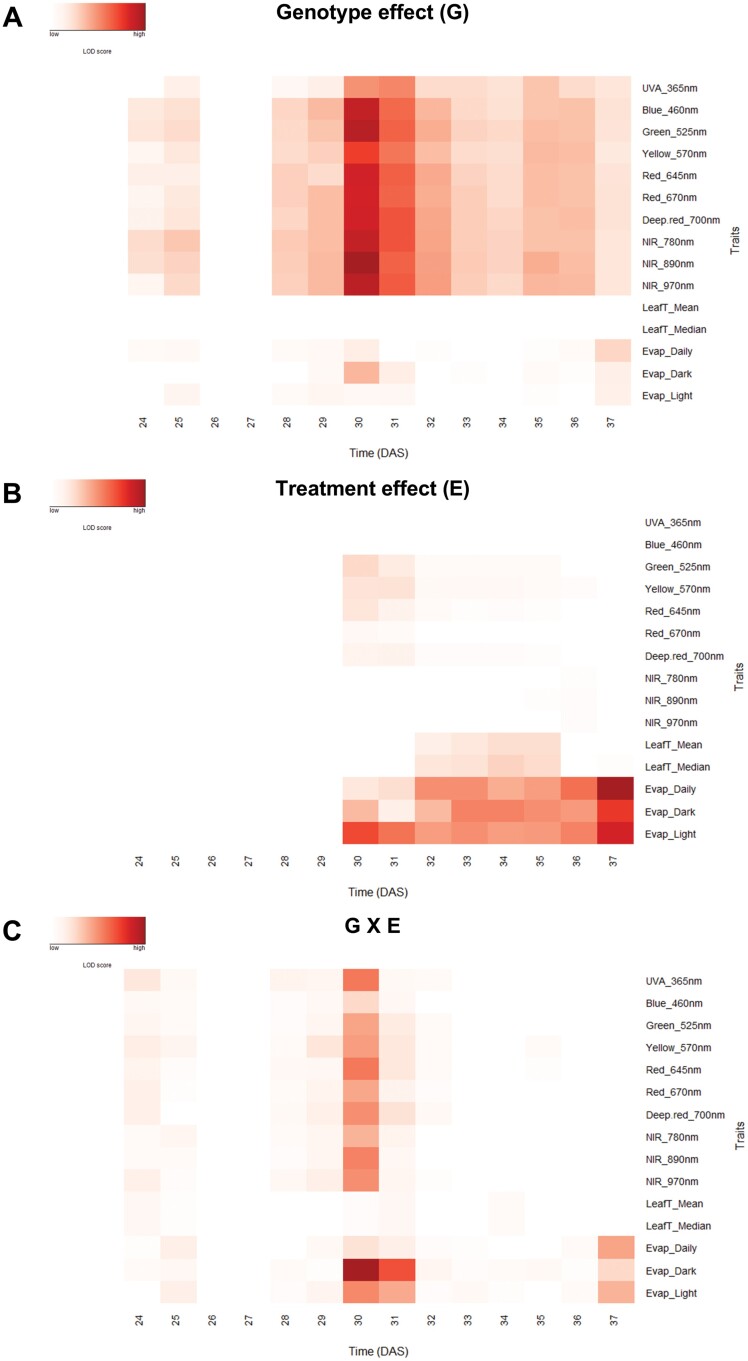
Phenotypic variation over time of wheat plants exposed to high temperature (WW38) or water deficit at high temperature (WD38). (A) Statistical significance of genotype effect, (B) treatment effect (WW38 versus WD38), and (C) their interaction effect (G×E) for each phenotype trait measured each day. The shaded plot indicates the significance level (Bonferroni-corrected *P*-values) in LOD scores (-log probability). There is no difference between the group means in white cells, and different shades of red indicate the strength of the significant difference between groups.

Traits with low reproducibility and high collinearity were filtered and extracted from the dataset to avoid redundant or low-quality descriptors of the 10 wheat genotypes (Table 1). A total of 15 traits reflecting the three trait classes were ­maintained (Fig. 1): two traits reflecting leaf temperature (LeafT), three traits associated with the evapotranspiration processes (Evap.), and 10 traits reflecting the multispectral signature. The statistical significance of phenotypic variance (LOD score) was determined to identify the traits that could reflect an effect of the genotype (G), treatment (E, environment), and their interaction (G×E) for each measurement day. All traits, except LeafT, increased LOD scores for the G effect when stress was imposed (30–32 DAS, Fig. 1A; Supplementary Table S1), but a gradual decrease of differences in multispectral traits was observed until the end of the experiment (37 DAS). Interestingly, at 30 DAS, it was possible to discriminate between different groups: check lines (BAJ, BORLAUG); PT lines (PASTOR, PUBWB, SOKWB_1, SOKWB_2); and the other genotypes (Supplementary Fig. S1A, B). At 37 DAS, the distribution of the genotypes was more uniform and centralized horizontally under WD38 (Supplementary Fig. S1C) and vertically under WW38 (Supplementary Fig. S1D). At this stage, most of the traits exhibited similar LOD scores (Fig.1A; Supplementary Table S1). The environment effect (E) altered thermal evapotranspiration progressively after stress imposition (Fig. 1B; Supplementary Table S2). However, a slower reaction was observed for LeafT that only changed after 31 DAS (Fig. 1B; Supplementary Table S2). Multispectral traits showed a distinct dynamic after stress imposition (Fig. 1B; Supplementary Table S2). No change was observed for UV-A and blue light (365–460 nm). On the other hand, green, yellow, and red light (525–700 nm) decreased LOD scores, showing a similar behaviour in G effect, and near infrared (NIR; 700–970 nm) LOD scores increased slightly after 35 DAS (Fig. 1B; Supplementary Table S2). All traits were observed to have significant G×E effects when the temperature increased from 25 °C to 38 °C (30 DAS; Fig. 1C; Supplementary Table S3). On the other hand, only the traits related to evapotranspiration expressed significant G×E differences until the end of the experiment (37 DAS), indicating a strong influence of high temperature and extended drought on genetic factors related to these traits.

**Table 1.** The 10 wheat genotypes used in this study

Genotype ID^a^	Cross name	Information	GID CIMMYT	Source (tested))
PASTOR	W15.92/4/PASTOR//HXL7573/2*BAU/3/WBLL1	PT line	5865676	CIMMYT (7th SATYN
SOKWB_1	SOKOLL/WBLL1	PT line	6056139	CIMMYT (7th SATYN)
BORLAUG100	BORLAUG100 F2014	Check	7806808	CIMMYT (7th, 8th SATYN)
CMH82	CMH82.575/CMH82.801	Parental	1187021	CIMMYT (Reynolds *et al.*, 2007)
PUBWB	PUB94.15.1.12/WBLL1	PT line	6056064	CIMMYT (8th SATYN)
SOKWB_2	SOKOLL/WBLL1	PT line	6056140	CIMMYT (8th SATYN)
SOKOLL	SOKOLL	Check Parental	3825355	CIMMYT (7th, 8th SATYN)
KSPA	KS940935.7.1.2/2*PASTOR	Parental	5865910	CIMMYT (Manès *et al.*, 2012)
BAJ	BAJ #1	Check	5106304	CIMMYT (7th, 8th SATYN)
PARAGON	CSW1724-19-5-68//Axona/Tonic	UK elite line	NA	LEC, UK (Correia *et al.*, 2021)

ID adopted for this study based on the cross name simplification

### Indicators of water status

**Fig. 2.**
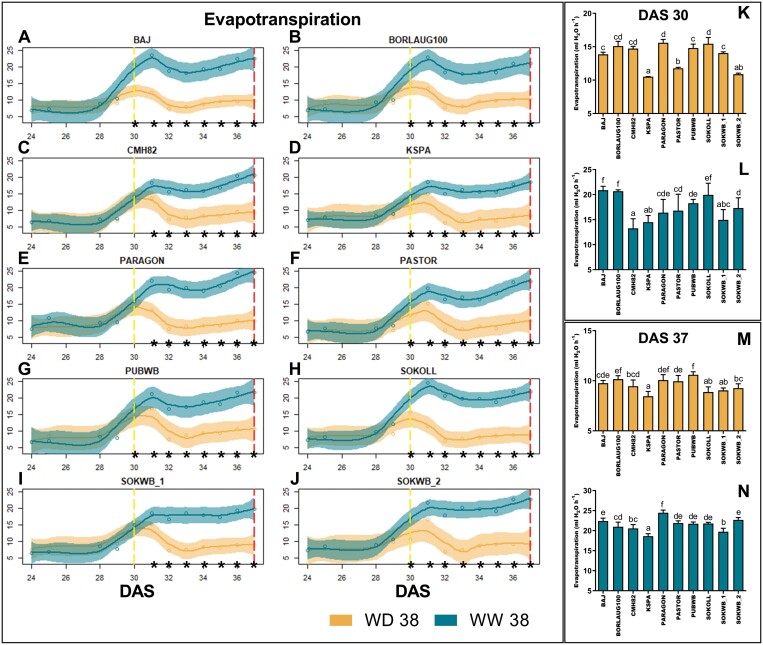
Evapotranspiration over time of wheat plants exposed to high temperature (WW38) or water deficit at high temperature (WD38). (A–J) Measurements were taken continuously throughout the experiment for each genotype in plants under WW38 and WD38. (K, L) Comparison of genotypes at the start of the stress imposition (30 DAS). (M, N) Comparison of genotypes at the end of the experiment (37 DAS). Blue lines and bars represent WW38 plants; yellow solid lines and bars represent WD38 plants. Shaded areas represent 95% confidence intervals. Yellow dashed lines denote stress imposition (30 DAS) and red dashed lines the end of the experiment (37 DAS). Asterisks denote significant differences between treatments in each genotype (*t*-test, *P*<0.05, A–J). Bar charts show mean values ±SEM (*n*=5 biological replicates). Different letters denote significant differences between genotypes (Duncan analysis, *P*<0.05).

G×E effects significantly changed the phenotypic variance of traits related to evapotranspiration over the period when plants were exposed to high temperature and water deficit. Daily evapotranspiration (Evap.daily) was explored in more detail to understand the relevance of these adjustments, generally related to the water status and transpiration efficiency. Evapotranspiration increased with temperature, although when plants were exposed to water deficit (WD38), water availability rapidly decreased and, under these conditions, evapotranspiration decreased after stress imposition in all genotypes (Fig. 2A–J). Only CMH82 (Fig. 2C), PARAGON (Fig. 2E), and SOKWB_1 (Fig. 2I) did not show a significant difference between treatments at 30 DAS. Under WD38 conditions (Fig. 2K), KSPA, PASTOR, and SOKWB_2 showed low evapotranspiration, while BORLAUG100, CMH82, PARAGON, and SOKOLL displayed high values. When only exposed to high temperatures (WW38, Fig. 2L), BAJ, BORLAUG, and SOKOLL showed the highest values, and CMH82, KSPA, and SOKWB_1 the lowest. At the end of stress exposure (37 DAS, Fig. 2M, N) under WD38 conditions (Fig. 2M), KSPA, SOKOLL, and SOKWB_1 showed low values of evapotranspiration, in contrast to PUBWB and BORLAUG100 with high values. Under WW38 (Fig. 2N), KSPA showed low levels of evapotranspiration and PARAGON showed high levels (Fig. 2N).

**Fig. 3.**
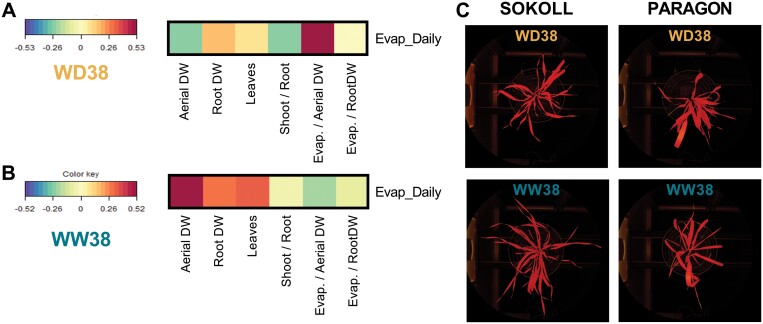
Relative importance of biomass and architecture traits to evapotranspiration at DAS 37 of wheat plants exposed to water deficit at high temperaturev (WD38, A) or only to high temperature (WW38, B). The heatmap represents a correlation between evapotranspiration and traits related to biomass and plant architecture. Evap, evapotranspiration; Evap/RootDW, evapotranspiration normalized to root dry biomass; Evap/AerialDW, evapotranspiration normalized to aboveground dry biomass; Shoot/Root, shoot to root mass fraction (Supplementary Fig. S2); Leaves, number of leaves per plant (Supplementary Fig. S2); Root DW: root dry biomass; Aerial DW, aboveground dry biomass. (C) Representative images of architectural differences between genotypes; segmented images of SOKOLL and PARAGON. The leaves are shown in red.

Under WD38, a positive correlation between the ratio of evapotranspiration to aboveground biomass (Evap/AerialDW) and evapotranspiration was observed (Fig. 3A). Under WW38, aboveground biomass correlated positively with evapotranspiration (Fig. 3B).

### Biomass prediction from images and plant growth modelling

**Fig. 4.**
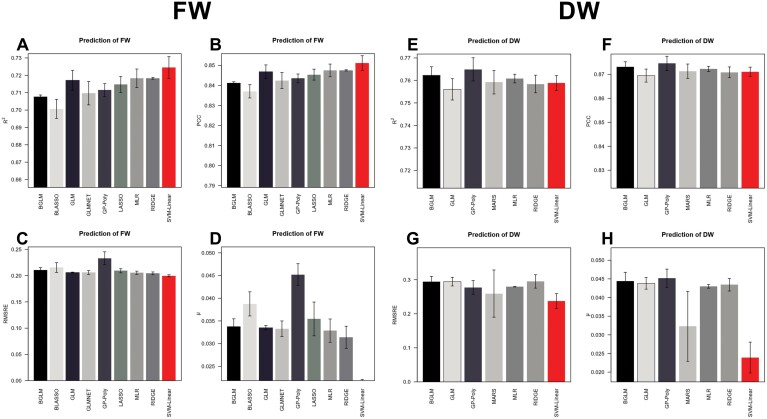
Performance of nine machine learning regression models considered for predicting plant biomass (FW, A–D, and DW, E–H) by image-extracted parameters (37 DAS). Models: (1) BGLM: Bayesian Generalized Linear Model; (2) BLASSO: Bayesian Lasso; (3) GLM: Generalized Linear Model; (4) GLMNET: Lasso and Elastic-Net Regularized Generalized Linear Models; (5) GP-Poly: Gaussian Process with Polynomial Kernel; (6) LASSO: Lasso Model; (7) MLR: Multivariate Linear Regression; (8) RIDGE: Ridge Regression; (9) SVM-Linear: Support Vector Machines with Linear Kernel. (A, E) *R*^2^, Pearson correlation coefficient of determination between the predicted values and the observed values; (B, F) PCC, Pearson correlation coefficient; (C, G) MRSR, root mean squared relative error of cross-validation; (D, H) μ, predictive bias between the predicted and observed values.

Regression models were developed to quantify the ability of geometrical traits to predict the aboveground biomass (DW, FW), measured at 37 DAS, in order to investigate the relationship between the image-extracted parameters and plant biomass. From the 16 tested machine learning regression models implemented in predMod (Chen *et al.*, 2018a), nine passed the defined criteria for model selection: *R*^2^>0.7; MRSRE <0.3; μ <0.05 (Fig. 4). Linear Support Vector Machine (SVM-linear, in red) was selected as the best model due to the low predictive bias (μ) and similar results for the other parameters (Fig. 4).

**Fig. 5.**
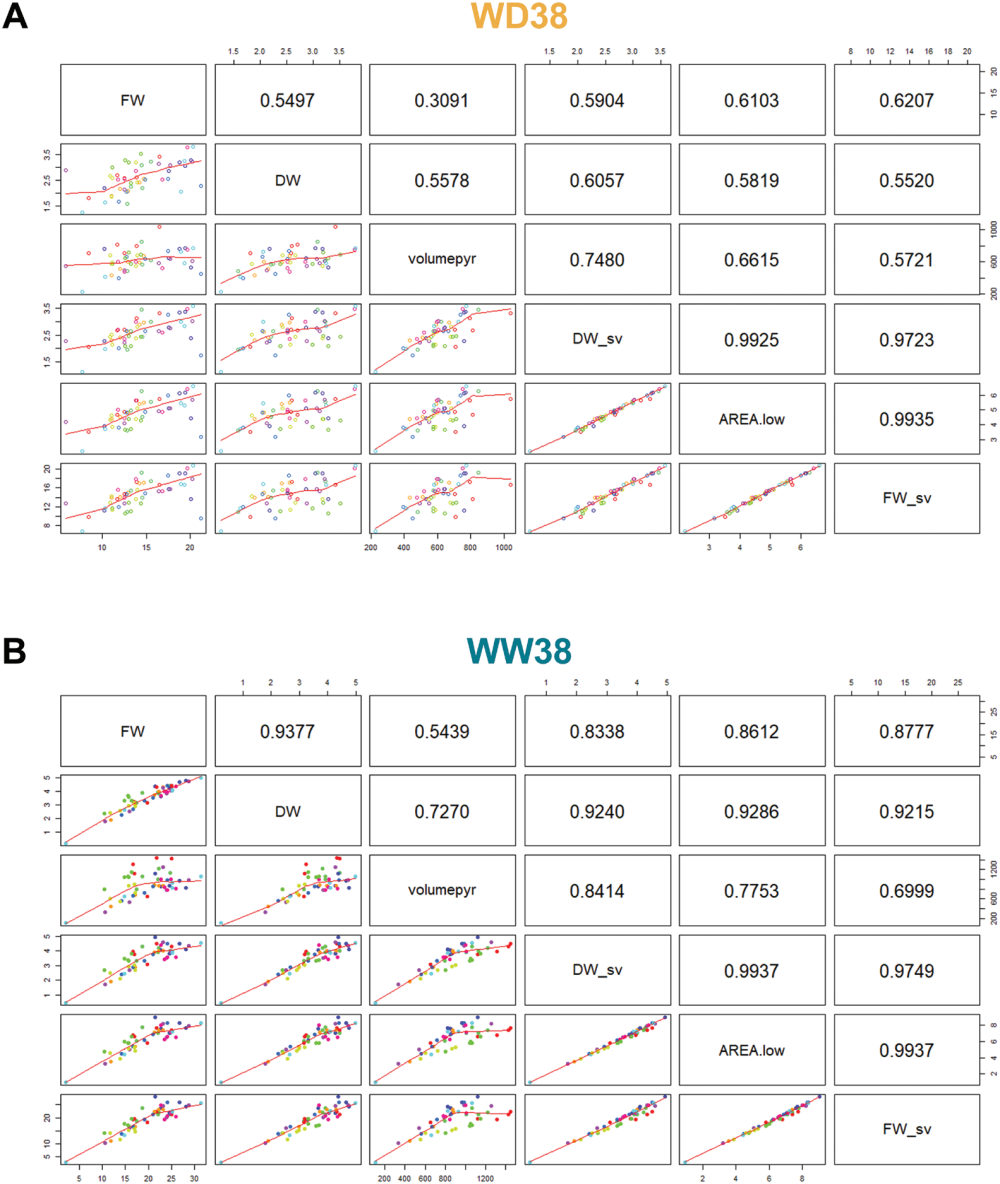
Pearson correlation matrix between manual measurements, image-derived features, and model-predicted data from plants growing at WD38 (A) and WW38 (B). Values are correlation coefficients (*r*). FW, aboveground fresh biomass; DW, aboveground dry biomass; FW_sv: predicted fresh biomass (SVM-linear); DW_sv, predicted dry biomass (SVM-linear); AREA.low, exposed leaf area extracted from images; volumepyr, plant pyramidal volume calculated based on image-derived features. Different coloured dots represent genotypes.

**Fig. 6.**
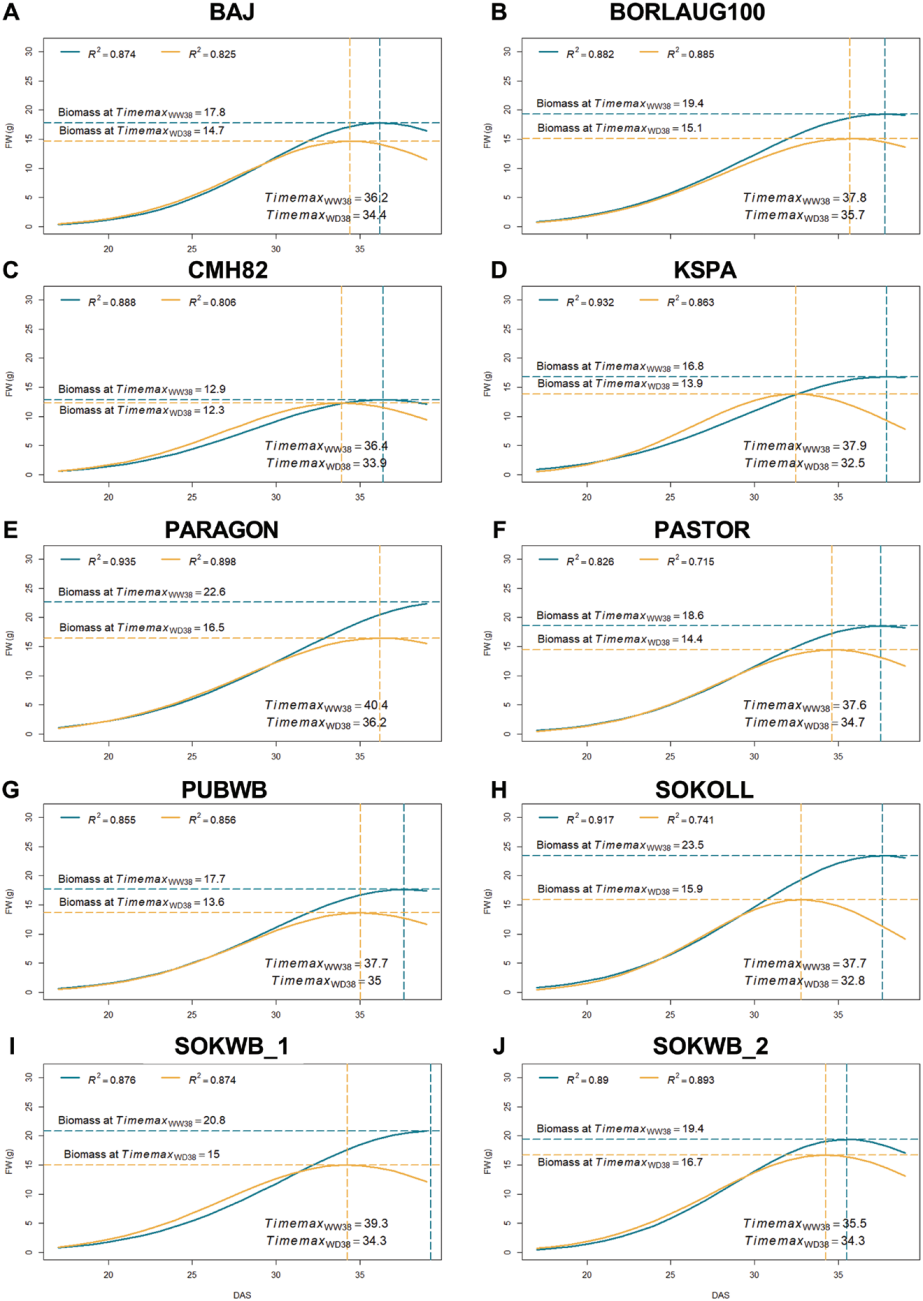
Modelling plant growth in 10 wheat genotypes exposed to high temperature (WW38) or water deficit at high temperature (WD38). Growth models are based on fitting predicted biomass values from 24 to 41 DAS using a bell-shaped model. Blue lines represent WW38 plants and yellow lines represent WD38 plants. Quality of fit (*R*^2^) of each model, the time point of maximum biomass (Timemax, vertical dashed lines), and maximum growth capacity (Biomass at Timemax, horizontal dashed lines) are indicated.

The geometrical trait area exposed (area.low) and pyramidical plant volume (volumepyr, calculated from the area and plant height) showed high predictive power. Moreover, the model constructed with the two parameters showed slightly higher accuracy to predict DW and FW, under WD38 and WW38 conditions, compared with the best single trait model, only with area.low (Fig. 5). FW predicted with the SVM-linear regression model built with the data extracted from images taken over plant growth (area.low and volumepy) showed the best correlation to FW measured and concomitant correlation to DW (Fig. 5). As the predicted fresh biomass (FW_sv) showed a better correlation to the measured biomass (FW and DW) compared with the predicted dry biomass (DW_sv), FW_sv was used as a proxy of plant aboveground biomass to model plant growth from 15 DAS until the forecast 41 DAS (Fig. 6).

To determine the best model for biomass accumulation in wheat plants subjected to WW38 and WD38, 10 different mechanistic models implemented in growMod (Chen *et al.*, 2018a) were tested. A bell-shaped model satisfied the previously established criteria (*R*^2^>0.7, *P*<0.05) for all genotypes in WW38 and WD38 conditions, with the best fit (*R*^2^) for all the genotypes under WD38 and comparable results with the logistic model in WW38, and was thus selected to represent plant growth (Fig. 6). Different dynamics were observed between genotypes and stress conditions. The maximum biomass prediction (Biomass at Timemax) under WW38 was extracted from the growth model of Sokoll at 37 DAS (23.5 g, Fig. 6H) and the lowest for CMH82 at 36 DAS (12.9 g, Fig. 6C). Under WD38, the highest value was observed in SOKWB_2 at 36 DAS (16.5 g, Fig. 6E) and the smallest in CMH82 at 35 DAS (12.7 g, Fig. 6C).

### Tolerance indices revealing stress symptoms

**Fig. 7.**
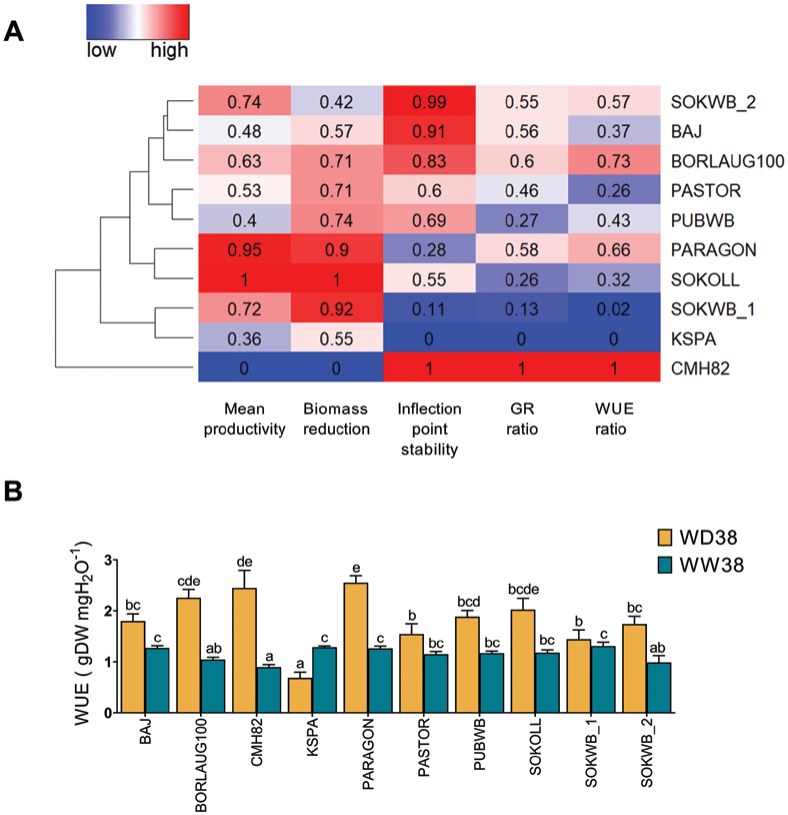
Stress tolerance indices of 10 wheat genotypes when exposed to high temperature (WW38) or water deficit at high temperature (WD38). (A) Heat map representation of rescaled values (0–1) of mean productivity, biomass reduction, inflection point stability, growth rate (GR), and water use efficiency (WUE) ratios of WD38 to WW38. Blue cells represent low values and red cells high values. Hierarchical clustering was applied to the matrix rows. (B) Water use efficiency, as biomass produced per water transpirated. Different letters denote statistically significant differences between genotypes (Duncan analysis, *P*<0.05). Mean productivity=(Biomass at Timemax_WW38_+Biomass at Timemax_WD38_)/2; Biomass reduction=Biomass at Timemax_WD38_/Biomass at Timemax_WW38_; Inflection point stability=Timemax_WW38_−Timemax_WD38_; GR ratio=GR_WD38_ GR_WW38_; WUE ratio=WUE_WD38_/WUE_WW38_.

To characterize and classify the genotypes according to their growth dynamics under WW38 and WD38, stress tolerance indices were calculated with the parameters extracted from the growth models and the genotypes clustered accordingly (Fig. 7). CMH82 showed a distinct behaviour, with high biomass stability, low productivity, minor changes in GR between conditions, and elevated WUE under WD38 (Figs 6C, 7). KSPA and SOKWB_1 showed a premature inflection point (Timemax) under WD38 relative to WW38, the highest reduction in the GR and WUE ratio (Figs 6D, I, 7). However, SOKWB_1 showed higher productivity and biomass reduction, whereas KSPA was the only genotype with a higher WUE under WW38 than under WD38 (Figs 6D, I, 7). PARAGON and SOKOLL revealed the highest mean productivity (MP) and high biomass reduction (BRR), although PARAGON showed a less accentuated reduction of GR and higher WUE under WD38 (Figs 6E, H, 7). BAJ, BORLAUG100, PASTOR, and PUBWB demonstrated a similar behaviour with average values (Figs 6A, B, F, G, 7); the only emphasis was on the higher value of WUE in BORLAUG100, due to the elevated productivity and inflection point stability (Figs 6B, 7). SOKWB_2 showed high MP, more akin to SOKWB_1 (Fig. 7). However, similarly to BAJ and BORLAUG100, it exhibited a high inflection point stability (Figs 6A, B, H, 7).

### Impact of WD38 and WW38 on phenotypic traits and adjustment of carbohydrate and antioxidant metabolism

**Fig. 8.**
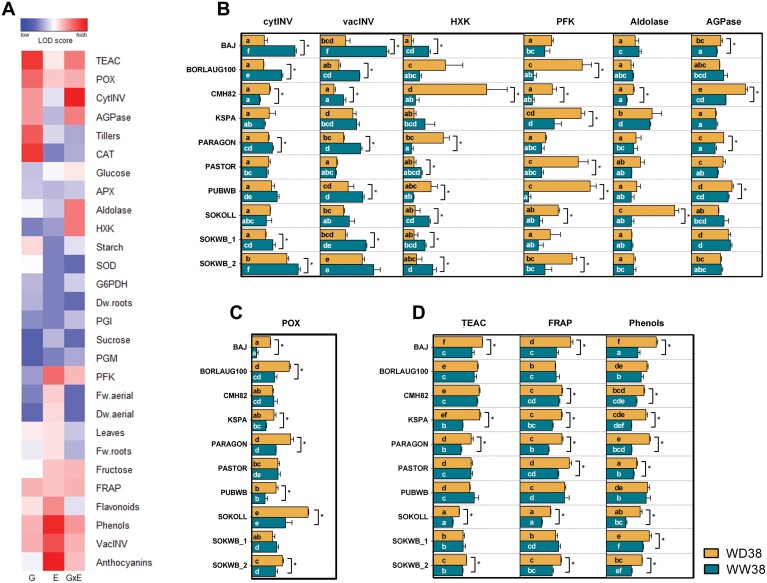
Adjustments of the carbohydrate and antioxidant metabolism of wheat when exposed to high temperature (WW38) or water deficit at high temperature (WD38). (A) Statistical significance of genotype effect, treatment effect (WW38 versus WD38), and their interaction (G×E) for each trait measured at the end of the experiment (37 DAS). The shaded plot indicates the significance level (Bonferroni-corrected *P*-values) in LOD scores (-log probability). Blue cells represent low LOD values and red cells high values. (B and C) Top 10 traits with the highest G×E LOD scores. (B) Carbohydrate metabolism enzyme activity expressed relative to the amount of TSP. (C) Peroxidase activity expressed relative to the amount of TSP. (D) Antioxidant capacities and total phenols (mg g^–1^ of equivalents). Asterisks denote significant differences between treatments in each genotype (*t*-test, *P*<0.05). Bars show mean values ±SEM (*n*=4–5 biological replicates), and different letters denote statistically significant differences between genotypes (Duncan analysis, *P*<0.05).

Significance of variance associated with genotype (G), treatment (E), and their interaction was evaluated for phenotypic traits including the activity of key enzymes and metabolites from carbohydrate and antioxidant metabolism to dissect the components relevant to the response to water deficit under high temperature. CAT, POX, cytINV, AGPase, and vacINV activities, antioxidant capacities (TEAC and FRAP), phenols, and starch content revealed the strongest G effects (Fig. 8A). On the other hand, anthocyanins, flavonoids, phenols, fructose content, vacINV and PFK activities, FRAP, and the number of leaves displayed the highest environmental effects (Fig. 8A). CytINV, vacINV, HXK, PFK, Ald, AGPase, and POX activities, TEAC, FRAP, and phenol content expressed significant G×E (Fig. 8A). The traits that expressed significant G×E differences at the end of the experiment were linked to carbohydrate and antioxidant metabolism and were explored in more detail to understand the regulatory mechanisms linked to WD38 and WW38. Except for KSPA, PASTOR, and SOKOLL, all the genotypes experienced changes in cytINV or vacINV activities. Of those, only CMH82 demonstrated an increase of cytINV activity under WD38. PUBWB only showed significant differences in vacINV and SOKWB_2 in cytINV, despite the high activity under both conditions in cytINV and vacINV (Fig. 8B). CMH82 showed the strongest activity of HXK under WD38 concomitantly with significant changes in PARAGON and PUBWB. Excluding KSPA and BORLAUG100, all the other genotypes exhibited significantly higher activity under WW38. BORLAUG100, CMH82, KSPA, PASTOR, PUBWB, SOKOLL, and SOKWB_2 demonstrated a significantly elevated activity of PFK under WD38. SOKOLL showed a robust activity of Ald under WD38, CMH82 exhibited differences in a minor scale, and KSPA revealed high intrinsic activity despite no differences between conditions. CMH82 showed elevated activity of AGPase under WD38 and, to a lesser extent, BAJ, PARAGON, and PUBWB showed a significantly higher activity under the same condition. On the other hand, significant increases in the antioxidant capacity and activity occur only under WD38 (Fig. 8C, D). BAJ, KSPA, PARAGON, SOKOLL, and SOKWB_2 exhibited changes in all the traits. BORLAUG only showed differences in POX activity and SOKWB_1 in phenol amount (Fig. 8C, D).

## Discussion

The impact of water deficit at high temperature on phenotypic traits related to reflectance proprieties, water use, and biomass accumulation was assessed in 10 spring wheat lines. The pool of genotypes with heterogeneous performance to drought stress or high temperature conditions was used to investigate G×E of trait responses in a phenotyping platform with a controlled high-temperature and water regime. Additionally, to understand the regulatory mechanisms of the primary carbohydrate and antioxidant metabolism associated with wheat response to drought at high temperature, the activities of key enzymes and metabolism associated with those pathways were analysed.

We identified an overall strong genotype reflectance signature throughout the experiment, which was intensified when temperature increased (30 DAS, Fig. 1A). This indicates constitutive differences in pigments/secondary metabolites and leaf structure between genotypes (Merzlyak *et al.*, 2003; Blackburn, 2007). On the other hand, only NIR intensities (700–970 nm) were related to the response to water deficit (LOD scores increased after 35 DAS, Fig. 1B), consistently with the decrease of water content, as reported in previous drought experiments (Seelig *et al.*, 2008; Chen *et al.*, 2014; Junker *et al.*, 2015). Together, these results showed that spectrometric measurements are important tools to detect the early response to the increase of temperature and to sense the response to drought stress. However, in our experimental set-up, it was not possible to discriminate the genotype response to the combination of high temperature and water deficit using only multispectral reflectance.

Moreover, G×E effects significantly changed evapotranspiration throughout the response to high temperature and water deficit (Figs 1C, 2, 3). At the end of the experiment under WD38, all the genotypes decreased evapotranspiration to 40% relative to WW38; however, we could identify three distinct transpiration behaviours in the face of water deficit at high temperature. While CMH82, PARAGON, and SOKWB_1 maintained the same evapotranspiration rate throughout the WD38 treatment, BAJ, BORLAUG, and SOKOL (check lines) decreased transpiration gradually upon stress imposition, and KSPA, PASTOR, PUBWB, and SOKWB_2 decreased transpiration on the first day under WD38 (70% relative to WW38), then maintained it stable for the following 2 d prior to a subsequent continuous decrease (Fig. 2). Additionally, the data establish a correlation under WD38 between evapotranspiration and transpiration efficiency (ratio between biomass and evapotranspiration) (Fig. 3A). On the other hand, evapotranspiration under WW38 was more related to aboveground biomass (Fig. 3B). Schoppach *et al.* (2016) also identified a trade-off between night and day transpiration and biomass in wheat response to high vapour pressure deficit. Other studies associated transpiration efficiency and increased yield potential in well-watered and water-limited environments with high stomatal densities and conductance (Roche, 2015; Shahinnia *et al.*, 2016). As water use is essential for either drought or heat tolerance, these results highlight the importance of the balance between evaporative cooling, water saving, and photosynthesis in wheat genotypes growing under water deficit and high temperature. Additionally, the results obtained in our experimental set-up show that gravimetric measurements are more accurate than thermal measurements in detecting the difference in the water status of genotypes in response to water deficit at high temperatures.

Biomass accumulation is a major indicator of plant performance and a key trait in plant breeding; however, conventional approaches are labour- and time-consuming, and plants need to be harvested and destroyed to measure biomass (Catchpole and Wheeler, 1992; Cobb *et al.*, 2013). Using imaging data and a linear support vector machine algorithm (SVM-linear), we could accurately estimate biomass accumulation under high temperature and the combination of high temperature and water deficit (Figs 4, 5). Furthermore, mechanistic models to estimate plant biomass are systematically and accurately used to estimate plant growth dynamics (Woodward and Hunt, 1983; Meade *et al.*, 2013; Tessmer *et al.*, 2013; Chen *et al.*, 2014). Our results indicate that a bell-shaped model is the best model to characterize wheat’s growth dynamic under single heat stress and when combined with water deficit (Fig. 6). Chen *et al.* (2014) also identified the bell-shaped model as the best model to describe the growth pattern of 18 barley genotypes in response to water deficit. The more accurate fit of this model, in contrast to a sigmoid model usually followed by plant growth under non-stressful conditions (Paine *et al.*, 2012; Tessmer *et al.*, 2013), can be explained by the more complex behaviour of plants growing under combined stress. Notably, plants under stressful conditions can undergo wilting, resulting in a decrease in volume. Accordingly, there was a higher correlation between FW and plant volume, as DW does not take into account changes in plant architecture due to water loss in the tissues. The decrease in FW and plant volume also increases the challenge of precise biomass prediction under water deficit. Future experiments including a greater number of destructive measurements of biomass will enable validation and improvement of plant growth prediction.

In this study, by the complementation of automatic non-invasive phenotypic measurements with robust methods for data extraction and analysis, we could characterize the complex and dynamic processes of wheat growth and water use (Fig. 7). Dissecting the growth dynamics and water use of the pool of wheat genotypes with divergent performance, we identified CMH82 as highly tolerant to WD38; however, this genotype also has a decreased potential to accumulate biomass under WW38, showing the lowest WUE and the worst mean productivity in both conditions. This behaviour would be advantageous under prolonged high temperatures associated with very dry conditions, but most probably would have a negative effect under more common agricultural conditions (Tardieu, 2012; Parent *et al.*, 2017). This tolerance mechanism was associated with a large modulation of carbohydrate metabolism, with higher activity of most enzymes related to sucrolytic and glycolytic pathways (Fig. 8). If carbohydrates are used for glycolysis in the leaves, sink strength is lost in other organs, and it is most likely that assimilates will be less available for reproductive organs and grain filling, causing yield penalties (Shokat *et al.* 2020; Roitsch and González 2004). Nevertheless, CMH82 showed higher activity of cytINV under WD38, which was previously associated with a faster recovery from water deficit and high temperature (Correia *et al.*, 2021). On the other hand, KSPA showed high WUE under WW38 but high susceptibility to WD38, without major changes in carbohydrate metabolism. These results suggest that modulation of carbohydrate metabolism upon water deficit at high temperature can be regarded as an essential protective mechanism. PARAGON showed the best trade-off between WUE under WD38/WW38 and biomass production, demonstrating tolerance to growth under high temperature and mid-water deficit generally experienced in field conditions. Most of the genotypes with high WUE under WD38 showed higher activity of HXK under this condition. HXK is responsible for the phosphorylation of hexoses, acting as a glucose sensor to interconnect nutrient, light, and hormone signalling networks for controlling growth in response to environmental variations (Moore *et al.*, 2003), and is associated with the acquisition of desiccation tolerance (Whittaker *et al.*, 2001). Almost all the genotypes demonstrated high activity of PFK related to the increase of glycolysis and reallocation of carbohydrates to respiration under WD38. The increase of cytINV and vacINV activities was also observed in leaves of barley exposed to high temperature (Cuesta-Seijo *et al.*, 2019), while tomato leaves exposed to water deficit demonstrated lower activity of cytINV and vacINV (Albacete *et al.*, 2015). Likewise, hexose accumulation was only observed in leaves under WD38 (Supplementary Fig. S3), showing that besides the increase of the sucrolytic activity under WW38, carbohydrates are being remobilized to other tissues.

Concerning the antioxidant enzymes evaluated, POX showed the highest genotype–environment effect and, although the different genotypes demonstrated different levels of activity, all of them showed increased POX activity under WD38. These results highlight the importance of POX activity in H_2_O_2_ scavenging under water deficit and high temperature, resulting in better control of ROS levels and protection against oxidative damage. Other studies also highlighted the importance of the modulation of POX activities to ROS scavenging under drought and high temperature (Koussevitzky *et al.*, 2008; Zandalinas *et al.*, 2017). ROS detoxification under WD38 was also associated with a non-enzymatic antioxidant defence, 0demonstrated by the increase of antioxidant capacity (FRAP and TEAC) and production of phenolic compounds.

In summary, this study illustrates the importance of canopy architecture to fine-tune transpiration required to achieve an equilibrium between water saving, leaf evaporative cooling, and biomass production when water deficit occurs at high temperature. Furthermore, it highlights the importance of transpiration efficiency for the maintenance of water uptake and transpiration/photosynthesis under these circumstances. The application of this methodology in further experiments connecting anatomical (number/size of stomata), phenological (number, shape, and angle of leaves), and functional traits (stomatal conductance and transpiration rate) to stress tolerance will help to elucidate the role of each component in the adaptation to fluctuations in water deficit associated with high temperature. Furthermore, our results highlighted the importance of adjustments in carbohydrate and antioxidant metabolism to tolerate these stressful conditions, more specifically in the sucrolytic (cytINV) and glycolytic pathways (HXK, PFK), and the ROS scavenging by POX and phenolic compounds. The integration of cell physiological phenotyping, via the semi-high-throughput determination of enzyme activity signatures and metabolites, with high-throughput phenotyping methods, proved to be an efficient approach to quantitatively characterize G×E of these complex traits. Further experiments with higher replication and collection of samples to quantify enzyme activity signatures/metabolites and biomass at different phenological stages can further help to prove the robustness of the provided approach and results. The application of this methodology for breeding programmes will facilitate the selection of promising candidates for wheat production in environments subjected to high temperatures and drought.

## Supplementary data

The following supplementary data are available at *JXB* online.

Table S1. LOD score table of genotype effects.

Table S2. LOD score table of treatment effect.

Table S3. LOD score table of G×E effect.

Fig. S1. Classification of wheat by multispectral signatures when exposed to high temperature or water deficit at high temperature.

Fig. S2. Aboveground architecture and root mass fraction of wheat when exposed to high temperature (WW38) or water deficit at high temperature.

Fig. S3. Impact of high temperature or water deficit at high temperature on the starch, sucrose, and hexose content of wheat leaves.

erac160_suppl_Supplementary_Table_S1-S3_Figure_S1-S3
