# Supplementary material for: High-throughput phenotyping of physiological traits for wheat resilience to high temperature and drought stress
Source: J Exp Bot. 2022 Apr 21;73(15):5235–51. doi: 10.1093/jxb/erac160 (PMC9440435; doi:10.1093/jxb/erac160)
Supplement: erac160_suppl_Supplementary_Table_S1-S3_Figure_S1-S3 [file erac160_suppl_supplementary_table_s1-s3_figure_s1-s3.pdf]

Supplementary data

Table S1 – Lod scores table genotype effect. Statistical significance of genotype effect for each phenotype trait measured in each day.

Genotype effect														
**Traits**	**LOD scores**													
**UVA_365nm**	1.8	10.8	0	0	6.9	11.6	35.2	38.5	19.1	18.7	16.7	24.8	19.1	15.7
**Blue_460nm**	13.7	17.2	0	0	20.7	27	64.1	44.7	27.8	19.8	17.8	24.6	25.6	16.8
**Green_525nm**	15.1	19.3	0	0	19.8	24.5	66.8	46	30	20.9	19.4	26.3	25.4	16.4
**Yellow_570nm**	8	14.5	0	0	19.1	21.8	53.5	42.1	26.3	19.2	18.2	26.6	26	14.2
**Red_645nm**	11.6	11.7	0	0	22	18.9	61.5	46.9	30.1	20.9	19	26	25.4	16.2
**Red_670nm**	8.8	13.9	0	0	21.9	25.9	60.8	45.8	29.7	22.4	19.1	25.9	25.4	16.8
**Deep red_700nm**	10.3	15.1	0	0	20.3	25.9	61	48.7	30.9	22.5	20.1	25.7	26	16.5
**NIR_780nm**	18.7	24.3	0	0	23	26.1	64.1	48.8	31.7	22.7	21	25.5	25.2	15.3
**NIR_890nm**	18.6	21.4	0	0	22.8	27	71.7	46.2	32.5	23	21.3	29.4	25.9	15.2
**NIR_970nm**	7.4	19.4	0	0	21.9	26.6	66	47.3	33.3	22.6	19.8	27.4	26.7	15.2
**LeafT_Mean**	0	0	0	0	0	0	0	0	0	0	0	1.9	0	0
**LeafT_Median**	0	0	0	0	0	0	0	0	0	0	0	1.8	0	0
**Evap_Daily**	4.4	5.7	0	0	4.4	6.4	12.3	0	2.3	1.4	1.4	2.4	4.3	20.7
**Evap_Dark**	1.7	2.1	0	0	1.7	5.3	27.8	12.5	0	2.7	1.9	4.3	2.2	11.7
**Evap_Light**	0	8.7	0	0	4.4	8.5	6.3	6.7	0	2	0	2.9	1.9	10.8
**DAS**	24	25	26	27	28	29	30	31	32	33	34	35	36	37

Note: If LOD = 0 the correspondent trait was not significant (adjusted $P<0.05$).

Table S2 – Lod scores table treatment effect. Statistical significance of treatment effect for each phenotype trait measured in each day.

Treatment effect														
**Traits**	**LOD scores**													
**UVA_365nm**	0	0	0	0	0	0	0	2.1	0	0	0	0	0	0
**Blue_460nm**	0	0	0	0	0	0	0	0	0	0	0	0	0	0
**Green_525nm**	0	0	0	0	0	0	22.7	15.5	5.5	4.6	4.8	4.4	2	0
**Yellow_570nm**	0	0	0	0	0	0	18.7	19.7	6.5	6.6	6.8	5.5	3.9	0
**Red_645nm**	0	0	0	0	0	0	18	12.1	4.3	3	3.3	3.1	0	0
**Red_670nm**	0	0	0	0	0	0	7.1	5.7	2	1.7	1.8	0	0	0
**Deep red_700nm**	0	0	0	0	0	0	11.2	11.7	3.5	3.3	3.7	2.7	1.4	0
**NIR_780nm**	0	1.4	0	0	0	0	0	0	0	0	0	1.4	2.6	2.1
**NIR_890nm**	0	0	0	0	0	0	0	0	0	0	0	3	3.4	2.3
**NIR_970nm**	0	0	0	0	0	0	1.6	0	0	0	0	2.3	3.3	2.2
**LeafT_Mean**	0	0	0	0	0	0	0	0	14	16.9	20.8	20.8	1.5	1.9
**LeafT_Median**	0	0	0	0	0	0	0	0	17.5	19.4	24.2	21.8	2.1	2.7
**Evap_Daily**	0	0	0	0	0	1.5	16.9	21	41.7	41.6	34	38.5	49.5	82.2
**Evap_Dark**	0	0	0	0	0	0	30.5	13.2	30.5	45	45.1	41.6	39	63
**Evap_Light**	0	0	0	0	0	0	58.9	49.1	38.5	41.6	37.8	39.1	45	69.3
**DAS**	24	25	26	27	28	29	30	31	32	33	34	35	36	37

Note: If LOD = 0 the correspondent trait was not significative (adjusted P<0.05).

Table S3 – Lod scores table GXE effect. Statistical significance of the genotype and treatment interaction effect for each phenotype trait measured in each day.

GXE effect														
**Traits**	**LOD scores**													
**UVA_365nm**	4.7	1.9	0	0	3.2	2.6	13.5	1.8	0	0	0	0	0	0
**Blue_460nm**	1.8	1.4	0	0	0	2	6.5	2.3	0	0	0	0	0	0
**Green_525nm**	2.4	1.4	0	0	0	2.5	10.5	4.4	0	0	0	0	0	0
**Yellow_570nm**	4.2	2.9	0	0	1.5	5.1	11	4.9	1.4	0	0	0	0	0
**Red_645nm**	3.1	0	0	0	1.9	2.2	13.5	4.9	0	0	0	0	0	0
**Red_670nm**	3.6	0	0	0	1.5	3.1	10.3	3.4	0	0	0	0	0	0
**Deep red_700nm**	3.6	0	0	0	1.8	3.6	11.8	5.5	1.8	0	0	0	0	0
**NIR_780nm**	1.4	2.6	0	0	1.4	2.8	9.3	3.3	0	0	0	0	0	0
**NIR_890nm**	0	1.6	0	0	0	2.8	13	2.2	0	0	0	0	0	0
**NIR_970nm**	3.6	0	0	0	2.3	3.8	12	2.7	0	0	0	0	0	0
**LeafT_Mean**	2.2	0	0	0	0	0	0	2.2	0	0	0	0	0	0
**LeafT_Median**	2.2	0	0	0	0	0	0	2.1	0	0	1.4	0	0	0
**Evap_Daily**	0	4	0	0	0	1.7	5.6	3.8	0	0	0	0	0	10.5
**Evap_Dark**	1.4	2.3	0	0	1.6	0	23.6	16.5	2.8	0	1.5	1.8	0	6.5
**Evap_Light**	0	3.8	0	0	0	3.1	12.4	10	0	1.7	0	0	1.4	9.3
**DAS**	24	25	26	27	28	29	30	31	32	33	34	35	36	37

Note: If LOD = 0 the correspondent trait was not significative (adjusted $P<0.05$).

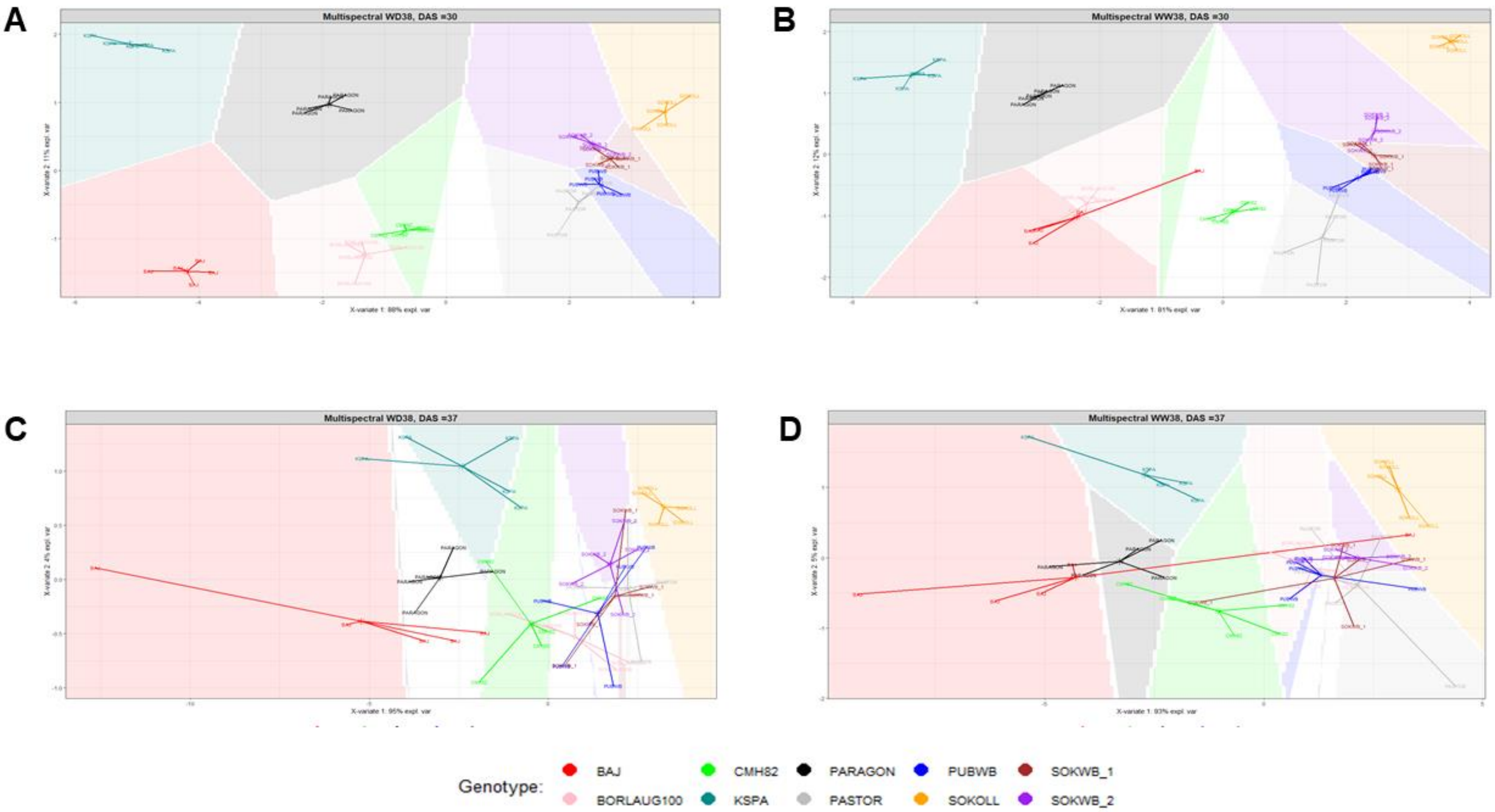

Figure S1 – Classification of wheat by multispectral signatures when exposed to high temperature (WW38, B and D) or water deficit at high temperature (WD38, A and C). (A-B) Partial least squares discrimination of wheat plants on the first day after stress imposition (30 DAS). **(C-D)** Discrimination at the end of the experiment (37 DAS). Background colours are predicted areas for the space representation of each genotype based on the centroid distance.

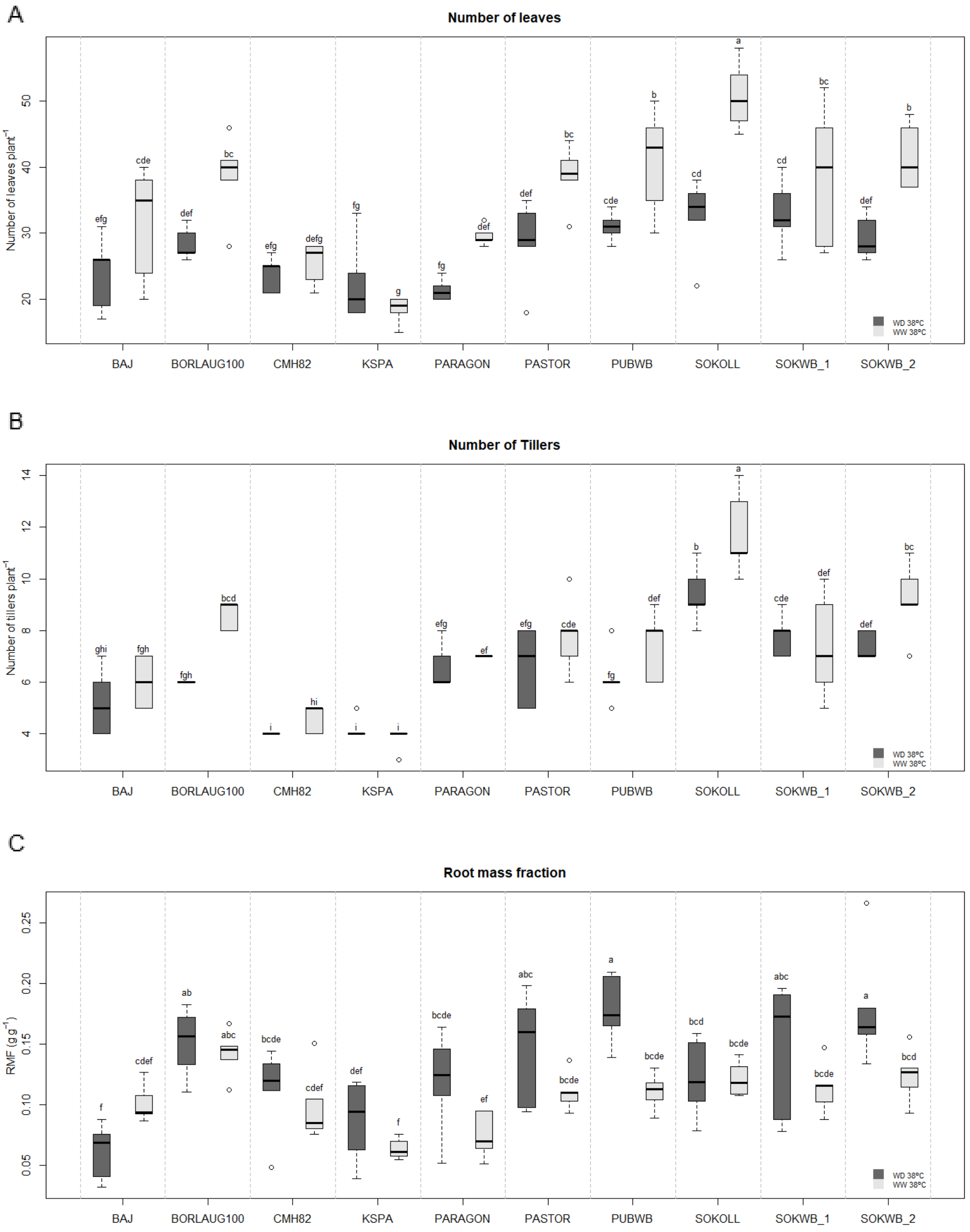

Figure S2 – Above ground architecture and root mass fraction of wheat when exposed to high temperature (WW38) or water deficit at high temperature (WD38). (A) The number of leaves. **(B)** The number of tillers. **(C)** Root mass fraction. Box plots show median values and interquartile range (IQR) (n = 5 biological replicates), and different letters denote statistically significant differences between genotypes (Duncan analysis, *P*<0.05).

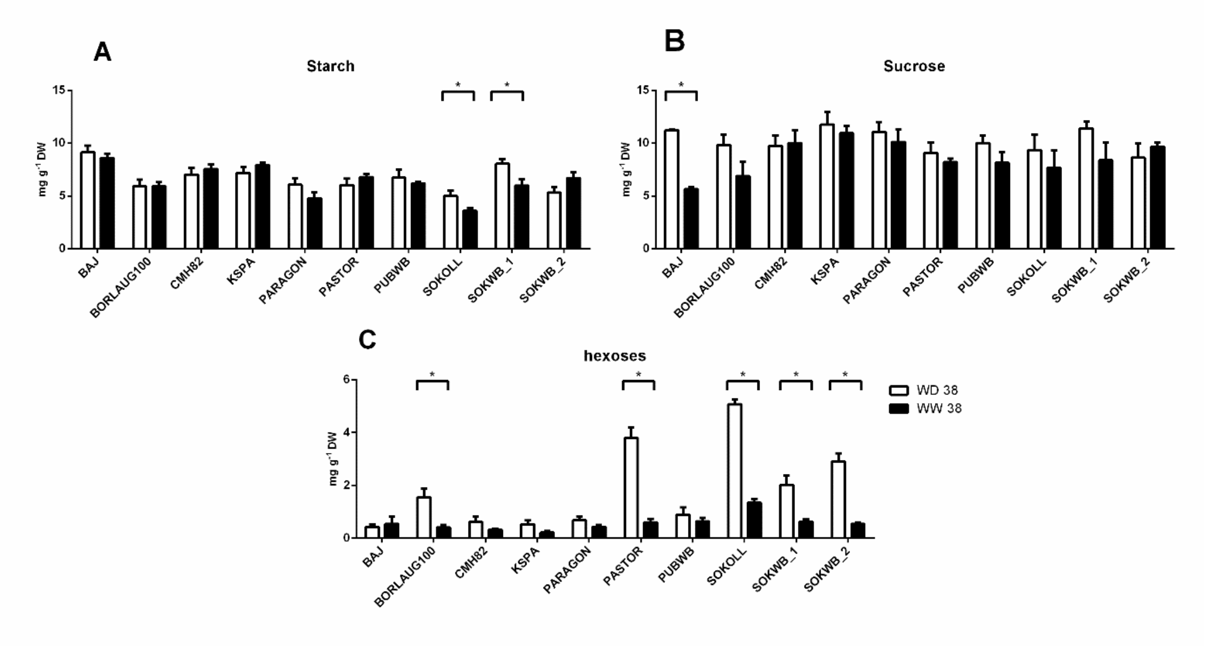

Figure S3 – Impact of high temperature (WW38) or water deficit at high temperature (WD38) on the (A) starch, (B) sucrose and (C) hexose content of wheat leaves. Values are means ± SEM (n = 5 biological replicates). Asterisks denote statistically significant differences between treatments in each genotype (t-test, P<0.05, A-C)
